# Home-Based Exergame Program to Improve Physical Function, Fall Efficacy, Depression and Quality of Life in Community-Dwelling Older Adults: A Randomized Controlled Trial

**DOI:** 10.3390/healthcare11081109

**Published:** 2023-04-12

**Authors:** Kyeongjin Lee

**Affiliations:** Department of Physical Therapy, College of Health Science, Kyungdong University, Wonju 24764, Republic of Korea; kjlee@kduniv.ac.kr

**Keywords:** exergame, older adults, physical function, fall

## Abstract

This study aimed to investigate the effects of home-based exergame programs on physical function, fall efficacy, depression, and health-related quality of life in community-dwelling older adults. Fifty-seven participants aged 75 years or older were divided into control and experimental groups. The experimental group received a home-based exergame program that included balance and lower-extremity muscle strength for 8 weeks. The participants exercised at home for 50 min three times a week and were monitored through a video-conference application. Both groups received online education on musculoskeletal health once a week, whereas the control group did not exercise. Physical function was assessed using the one-leg standing test (OLST), Berg balance scale (BBS), functional reaching test (FRT), timed up-and-go test (TUGT), and five-times sit-to-stand (FTSTS). Fall efficacy was assessed using the modified falls efficacy scale (MFES). Depression was assessed using the geriatric depression scale (GDS). Health-related quality of life was assessed using a 36-item short-form health survey (SF-36). The experimental group showed an overall improvement in OLST, BBS, FRT, TUGT, and FTSTS (*p* < 0.05). MFES was significantly increased in the experimental group after the intervention (*p* < 0.05). The GDS significantly decreased in the experimental group after the intervention (*p* < 0.05). In SF-36, role limitations due to physical health, general health, and fatigue (energy and fatigue) items improved in the experimental group after intervention (*p* < 0.05). An 8-week home-based exergame program improved physical function, fall efficacy, depression, and health-related quality of life in older adults. The study was registered on ClinicalTrials.gov (NCT05802537).

## 1. Introduction

Population aging presents a major problem for society [1]. As the older adult population, which accounted for 17% of the population in 2020, is expected to increase to 22% by 2040, population aging is expected to accelerate even further [2]. To manage an older population, healthy aging must be ensured, and the first priorities are physical activity and sarcopenia prevention [3,4].

Aging and inactivity are associated with loss of muscle mass, structure, and strength [5]. For individuals over 50 years of age, the muscle loss rate is 1–2% per year [6]. Sarcopenia, defined as a decrease in skeletal muscle mass and a decline in muscle strength and physical function due to aging, presents a challenge for healthy aging [4].

Furthermore, the recent prolonged COVID-19 pandemic has adversely affected the health of the older adult population [7]. As part of the global COVID-19 mitigation policies, control measures were implemented, such as community closures and social distancing [8,9]. This reduced social activities, including sports and leisure activities, which in turn reduced the amount of physical activity in older adults and accelerated the aging process [7,10].

Decreased physical activity is closely related to an increased risk of developing diseases, such as depression, diabetes, coronary artery disease, and poor cardiorespiratory function [11,12]. Muscle loss can lead to weakness in physical function, movement disorders, decreased balance and walking ability, and falls [13]. The older adult community are also more prone to suffer from physical injuries, such as fractures due to falls, which is a major cause of depression and death [14,15,16].

In order to maintain good health and reduce the risk of falls, older individuals need to engage in regular physical exercise [17,18]. Specifically, exercises that target leg strength and balance effectively improve postural control and reduce the likelihood of falls [17]. In addition, regular exercise can have a positive impact on cognitive function. It may help prevent or alleviate depressive symptoms, leading to an overall improvement in well-being and quality of life [19]. Given the many benefits of physical exercise for successful aging, it is recommended that healthcare professionals encourage elderly patients to incorporate exercise into their daily routines as a preventative measure [20]. When facing the current challenges caused by COVID-19 regulations, regular physical activity is important for maintaining good physical and mental health [10]. However, public health agencies have discouraged seniors, who are considered a risk group, from exercising in gyms, plazas, and other places when social distancing is recommended [8,9]. Few alternatives have been offered to these individuals to maintain regular physical activity.

Home-based exergame programs are recreational video games that combine gameplay with exercise and are gaining popularity as a way to encourage participation in physical activity at home [20,21]. Home-based exergame programs have been shown to encourage physical activity and induce light-to-moderate intensity movements by actively engaging participants in the game [20]. Particularly during the COVID-19 pandemic, home-based exergame programs ease at-home training and are becoming increasingly popular [22]. In addition, they have the advantage of easy accessibility because they are economical and easily distributed, and the exercise intensity can be adjusted according to the individual’s physical strength without the supervision of a physical therapist [22]. The game element of exergame programs provides various types of motivating feedback, such as encouraging commentary, bonuses, and music to create an interactive and exciting experience [20,21,22,23]. In previous studies, various games and types of exercises were performed using the Nintendo Wii or Xbox Kinect platforms, and showed improved balance, cognitive function, and daily living activities in older adults [23,24]. Exergames are increasingly being used as an alternative to traditional rehabilitation-based exercise programs to improve daily activity levels and fitness in older individuals [20,21,23,24].

These programs have been widely used as evaluation or rehabilitation tools for individuals with cognitive impairment due to neurological diseases, such as Parkinson’s disease and stroke, and patients with chronic back pain; however, older adults often have difficulties in using technology [25,26,27]. The purpose of this study was to investigate the effect of a home-based exergame program on physical function, fall efficacy, depression, and quality of life in older adults. For the purpose of the study, we established the following hypotheses. Older adults who participate in a home-based exergame program will experience significant improvements in physical function, fall efficacy, depression, and quality of life compared to those who do not participate in the program.

## 2. Materials and Methods

### 2.1. Study Design

This study was designed as a single-blind (rater) randomized controlled trial with an 8-week intervention period. The Ethics Committee of Kyungdong University approved the study protocol in accordance with the Declaration of Helsinki and all experiments were performed in accordance with these guidelines and regulations. After being provided with a complete description of the study, all participants signed a written consent form prior to the initiation of the study.

### 2.2. Participants

Participants were 75 years or older, registered at the S Senior Welfare Center in Seoul, Korea, and were recruited with the use of an advertisement in a fall-prevention exercise club. The inclusion criteria were the ability to walk independently with or without a walking aid and a mini-mental state examination score ≥ 24. Exclusion criteria were as follows: musculoskeletal disorders such as fractures or dislocations; neurological impairment or mental disorders; and uncontrolled endocrine, cardiovascular, or urinary system diseases.

Of the 92 individuals recruited using the advertisement, 17 were excluded according to the criteria and 15 refused to participate. The remaining 60 participants were randomly assigned to the experimental or control group. Selection bias was minimized using Random Assignment Software (version 2.0) (M. Saghei, Isfahan, Iran) [28]. The ratio of the participants in the two groups was 1:1. Numbers were allocated according to the registration numbers at the welfare center. An individual, independent of the study, performed the randomization procedure.

### 2.3. Sample-Size Calculation

To calculate the sample size, G-power 3.19 software (Heinrich Heine University Düsseldor, Düsseldorf, Germany) was used with a significance level of 0.05 and a power of 0.8. The effect size was calculated as 0.78, which was based on the balance variables from a pilot study. The sample size per group was 27, and after considering dropouts, 30 subjects were recruited per group.

### 2.4. Experimental Procedures

The 60 participants completed a demographic questionnaire and underwent a pre-test in the exercise program room at the S Senior Welfare Center. For the pre-test, the one-leg standing test (OLST), Berg balance scale (BBS), functional reaching test (FRT), timed up-and-go test (TUGT), and five-times sit-to-stand test (FTSTS), which correspond to physical function evaluations, were performed. The modified falls efficacy scale (MFES) was used to confirm fall efficacy. Depression was measured using the geriatric depression scale (GDS). The 36-item short-form health survey (SF-36) was used to measure health-related quality of life. After the pre-test, participants in the experimental group performed a home-based exergame program for 50 min three times a week for eight weeks, and the control group was without intervention. Eight weeks later, the post-test was conducted in the same manner as the pre-test.

After the pre-test, the exercise program methods and procedures were explained, and the participants were shown how to operate the video game systems. Guidelines for the systems were distributed and guardians were asked to participate in the initial education. For the participants who did not partake in the initial education, helpers provided visiting education for several weeks to ensure their familiarization with the game. Participants who discontinued the program due to changes in their health status during the intervention period and those who participated in <80% of the total program were excluded from the final analysis. In the experimental group, two participants were excluded due to illness. In the control group, one participant who moved to another area was excluded.

**Figure 1:**
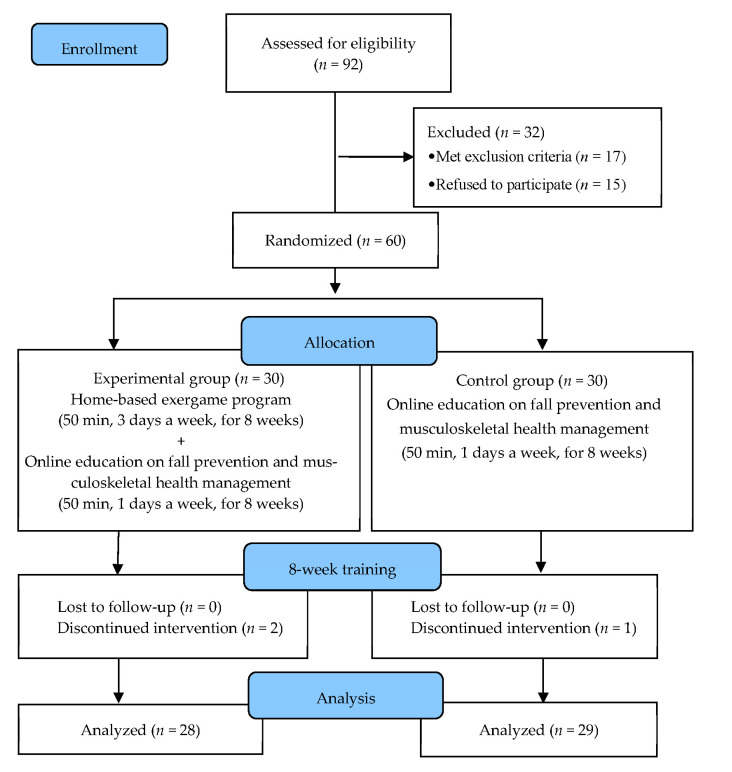
Flow diagram of the study.

Finally, 28 participants from the experimental group and 29 from the control group participated in this study. All participants underwent the pre- and post-test, and the data from these tests were analyzed (Figure 1).

### 2.5. Intervention

The experimental and control groups participated in online education on fall prevention and musculoskeletal health management for 50 min once a week for eight weeks [29]. Online training was conducted using a video-conference application. The training contents included education on aging and musculoskeletal disorders, fall prevention strategies, and falls and fractures under the instructor’s guidance.

The home-based exergame program was conducted at the participants’ homes for 50 min, three times a week, for eight weeks. The Nintendo game console (Switch, Nintendo, Kyoto, Japan) and Ring Fit Adventure program (Switch, Nintendo, Kyoto, Japan) were used. The Ring Fit Adventure program uses Ring-Con and Joy-Con to monitor body movements. Ring-Con is a circular controller with a diameter of approximately 30 cm and is constructed of elastic material. It is used as an armed sensor by attaching the Joy-Con (R) to it and is equipped with a precise force sensor that can recognize tightening and pulling movements. In addition, the movement of each body part is reflected in the game using Joy-Con’s acceleration and gyro sensors mounted on the Ring-Con and leg strap. Among the games available on the Ring Fit program, we selected lower-extremity muscle strength and balance games. The final decision was made by the collective opinion of experts in older adult exercise dynamics.

The exergame program consisted of a 10 min warm-up, 30 min of exercise, and a 10 min cool-down. The warm-up exercise consisted of stretching using the Ring Fit program and a leg massage using a massage ball. For the exercise portion, the adventure mode was selected. The participants performed yoga to increase balance, and leg and abdominal exercises to strengthen the lower-extremity muscles. Ring Fit adventure involves exploring more than 20 different worlds and using real-life exercises to defeat the bodybuilder dragon and his minions. Jogging in place to cross grassy plains and climb stairs is expected to strengthen the lower extremities’ strength and improve the participants’ dynamic balance ability. Exercises to defeat the bodybuilder dragon and his subordinates consist of strength training of the arms, abdomen, and legs and yoga exercises to improve balance. Leg exercises include squats, knee lifts, thigh presses, wide squats, and side steps. Abdominal exercises include knee to chest, plank, leg raise, and seated ring raise. Arm exercises include overhead press, front press, bow pull, triceps kick back, and back press. Yoga exercises include warrior 1 pose (lunge pose), revolved crescent lunge pose, warrior 2 (wide stance pose), and warrior 3 pose (single-leg stance pose). As players complete a level, their score increases, and the game’s difficulty gradually increases. Participants are designed to perform various exercises while immersing themselves in virtual reality. The cool-down portion consisted of stretching and breathing exercises. Prior to commencing the exercise regimen, the researcher ensured that the home environment was suitable for physical activity by checking the distance from the TV and securing the exercise area. In addition, the researcher installed console devices at each elderly participant’s home and provided instructions on how to use them to both the participants and their family helpers. Finally, the researcher laid a non-slip mat on the floor and explained how to perform the exercises. If fatigue, pain, shortness of breath, or dizziness were present, participants were asked to stop exercising and contact the researcher. Finally, the researcher asked participants to report by phone or text message after the exercise.

Participants in the control group were not provided with any exercise instructions for eight weeks, and no exercise program that could affect post-test results was implemented.

### 2.6. Outcome Measurements

#### 2.6.1. Physical Function

The participants’ physical function was evaluated in terms of balance ability and lower-extremity muscle strength. Balance ability was assessed using the OLST, BBS, FRT, and TUGT. The FTSTS was used to evaluate lower-extremity muscle strength.

The OLST was used to evaluate static postural balance [30]. The OLST is a simple, reliable, and valid test for assessing balance and fall risk in older adults [31]. Each participant was asked to keep their eyes and arms open for as long as possible, and the dominant leg was used. The time for the opposite foot to touch the ground was measured in seconds using a stopwatch (HS-3V-1B, Casio, Tokyo, Japan). This test was performed three times, and the highest score was recorded [32].

To evaluate dynamic postural balance, the BBS was used. It has a perfect score of 56 and consists of 14 items, with a perfect score of 4 for each item. The BBS has been found to have high reliability and internal validity for measuring balance ability, with correlation coefficients of 0.99 and 0.98, respectively [33].

The FRT was used to evaluate movement limits. This test assesses the limits of physical stability and measures dynamic balance and flexibility while performing functional tasks. The FRT has high evaluation–revaluation reliability and inter-measurement reliability, with r values of 0.89 and 0.98, respectively [34]. The participants were asked to stand with their feet shoulder-width apart and raise their arms parallel to shoulder height while extending their arms as far as possible. While standing, the distance between the length of an outstretched arm in a maximal forward reach was measured, while maintaining a fixed base of support. The results are presented as the average of three consecutive measurements.

The TUGT was used to assess functional movement and mobility. It is used to evaluate the risk of falls and has a high ICC score of 0.99 [35]. This test measures the time it takes for an individual to stand up in a standard armchair (seat height approximately 46 cm), walk a distance of 3 m, turn around, return to the chair, put on regular shoes, and sit down [35]. It was conducted without an auxiliary tool. The time was measured using a stopwatch, and the average was taken after three measurements.

The FTSTS was used to evaluate lower-extremity muscle strength. This test has good inter- (*r* = 0.94) and intra-rater (*r* = 0.99) reliabilities [36]. For the test, the subjects were instructed to sit in a chair without armrests, cross their arms over their chest, and then sit down and stand up as quickly as possible for five repetitions. The participants performed two tests. The time was measured using a stopwatch and averaged after three measurements.

#### 2.6.2. Falls Efficacy

The MFES, which was developed by Hill et al. [37] for older adults specifically, was used to evaluate the fall efficacy. The questionnaire consists of 14 questions to measure how confidently one could perform each motion without falling. Each item ranges from unconfident (0 points) to very confident (10 points). It is a 10-point scale, and the average was obtained by adding the scores of each question. The higher the score, the higher the confidence not to fall. The test–retest reliability of MFES was 0.80, showing good reliability, and Cronbach’s alpha coefficient for the MFES was 0.87, indicating good internal consistency [38,39].

#### 2.6.3. Depression

Depression was measured using the GDS-15, which comprises 15 questions and a higher score amounts to a higher depression score [40]. The GDS-15 is a reliable and valid measure for assessing depressive symptoms in older adults [41]. The GDS-15 is a frequently used depression screening tool and uses a yes/no format, which allows easy response for older adults. Unlike other assessment tools, the GDS does not include items related to physical symptoms.

#### 2.6.4. Health-Related Quality of Life

Health-related quality of life was measured by the SF-36 which allows participants to evaluate their general health status. The SF-36 is a reliable and valid measure of health-related quality of life that has been widely used in research and clinical practice [42,43]. The SF-36 covers physical function, bodily pain, role limitations due to physical health and emotional problems, mental health, social function, vitality (energy and fatigue), and general health (eight items), which are evaluated with a total of 36 items. Each item is scored on a Likert scale ranging from 1 to 5. Each item was summed according to the method suggested by Ware and Sherbourne [44], and the summed scores were converted into a score out of 100. Higher scores indicate better health.

#### 2.6.5. Mini-Mental State Examination (MMSE)

The cognitive ability of the participants was assessed by means of the MMSE as part of the subject selection process. The MMSE is a cognitive assessment tool that measures multiple cognitive functions and has demonstrated high reliability and validity in detecting moderate to severe dementia. The MMSE evaluates the participant’s orientation, registration, attention, calculation, recall, language, and copying. It is a quick and straightforward test that can be administered without any additional equipment [45].

### 2.7. Statistical Analysis

The Statistical Package for Social Sciences (SPSS version 24, IBM, Armonk, NY, USA) was used for all statistical analyses. For data normality, the Shapiro–Wilk test was used to confirm that the normality assumption was satisfied. Independent *t*-tests and chi-square tests were used to test the homogeneity between the two groups. The changes in the dependent variables were analyzed with a paired *t*-test, and the effect between groups was calculated using an independent *t*-test. Effect sizes were calculated to assess the strength of the training effect, and the minimal detectable change (MDC) and 95% confidence intervals (CIs) were calculated to reflect actual changes, in addition to errors. The statistical significance level (α) was set at *p* < 0.05.

## 3. Results

### 3.1. General Characteristics of the Subjects

There was no statistically significant difference in any data between the experimental and control groups in the pre-test, indicating homogeneity. After two participants in the experimental group and one in the control group dropped out, the data of the 28 participants in the experimental and 29 in the control group were statistically analyzed (Table 1).

### 3.2. Changes in Physical Function

Table 2 shows the results of the physical function according to the home-based exergame program. In the experimental group, there were statistically significant increases (*p* < 0.05) in the OLST, BBS, FRT, TUGT, and FTSTS after intervention. However, in the control group, the difference before and after the experiment was insignificant. The difference between the groups according to the experimental method showed a significant increase in the experimental group (*p* < 0.05).

### 3.3. Changes in Fall Efficacy and Depression

Before the home-based exergame program, the MFES results of the experimental and control groups seemed identical. However, after the home exergame program, the experimental group showed an increase, while the control group showed a decrease, confirming a significant difference between the two groups (*p* < 0.05). Furthermore, before training, the two groups had identical GDS scores, and after training, a significant difference between the two groups was confirmed (Table 3).

### 3.4. Changes in Health-Related Quality of Life

Differences in SF-36 after the home-based exergame program were confirmed for each item. A significant increase was confirmed in the physical function item of the experimental group during the pre-test (*p* < 0.05), whereas no difference was found in the control group. The difference between the two groups was statistically significant (*p* < 0.05). In the role limitations due to physical health items, there was a significant increase in the experimental group (*p* < 0.05), but no significant change in the control group. The difference between the two groups was statistically significant (*p* < 0.05). No significant change was observed in the body pain item in the experimental and control groups, and no difference was found between the two groups. In the general health category, the experimental group showed a significant increase (*p* < 0.05), whereas no change was noted in the control group. The difference between the two groups was statistically significant (*p* < 0.05). The energy and fatigue items increased significantly (*p* < 0.05) in the experimental group but were maintained in the control group. The difference between the two groups was statistically significant (*p* < 0.05). In social functioning, role limitations due to emotional problems, and mental health, there were no differences before and after the intervention in both the experimental and control groups (Table 4).

## 4. Discussion

The COVID-19 pandemic has brought many changes to society, and the impact of these changes continues to be evident. Owing to the phenomenon of avoiding public spaces, it is necessary to manage health at home [10]. In this study, a home-based exergame program was implemented in the older adult community to confirm its physical and mental effects and to propose a new exercise method in the post-COVID-19 era.

The impact of older adults’ balance and walking ability on falls is a critical area of research. As individuals age, changes in the musculoskeletal, neural, and sensory systems can lead to impaired gait and balance, which may contribute to falls and reduced function [46]. According to a study by Maki et al. [47], poor balance and gait were among the most significant risk factors for falls among older adults. Lord et al. [48] also found that a decline in walking ability, as measured by gait speed, was associated with an increased risk of falls. Furthermore, a study by Tinetti et al. [49] demonstrated that impairments in balance and gait were predictive of future falls among older adults. Impaired gait and balance are associated with reduced function in the elderly population, including decreased mobility, decreased independence, and decreased quality of life [46]. Reduced mobility can limit an individual’s ability to perform daily activities such as walking, climbing stairs, and getting up from a chair. Decreased independence can result in increased reliance on caregivers or family members, which can have social and emotional consequences [50]. Decreased quality of life can result from decreased participation in social and recreational activities, leading to social isolation and depression [46,51].

More recent studies have also investigated the relationship between balance, walking ability, and falls in older adults. Previous studies have shown that poor balance is associated with an increased risk of falls in older adults, and that gait speed significantly predicts falls in older adults with cognitive impairment [13,52]. Numerous studies have demonstrated that poor balance and gait impairments increase the risk of falls among older adults, and interventions to improve these factors can effectively reduce the incidence of falls [29,52]. These findings suggest that promoting balance and walking ability in older adults is essential to fall prevention strategies.

The 8-week home-based exergame program was found to have a positive effect on the physical function, fall efficacy, depression, and health-related quality of life in older adults. As for physical function, the effects on static balance, dynamic balance, and lower-extremity muscle strength were confirmed, and all variables showed significant improvement after the exercise program. Older adults show rapid muscle loss and loss of balance ability, which negatively affect the nervous, cardiorespiratory, and musculoskeletal systems [4,6]. Falls are a cause of rapidly deteriorating health in older adults; therefore, it is very important to prevent and manage falls [13,14]. In this study, Nintendo’s Ring Fit Adventure, used for exergames, combines game elements and exercise elements well, providing exercise programs such as improving lower-extremity muscle strength, balance, and core strengthening, which are closely related to falls. During the exergame program, motivation is induced by achieving the goal required in the game and providing a suitable exercise through difficulty adjustment. Cueing is a very important factor in exercise, and by appropriately assigning cueing in the game, the exercise is made more fun and the player feels motivated. A previous study on the balance and gait of older adults using the Nintendo Wii Fit board showed results similar to those of this study [23]. These studies have reported the effectiveness of video games for fall prevention and musculoskeletal health management in older adults [23,24,27].

In this study, it was found that balance ability improved in the experimental group, with significant improvements in the OLST, BBS, FRT, and TUGT. In the case of the OLST, the results of this study showed that balance ability improved by 30.3% in the experimental group. This can be explained by the fact that the repeated movements of standing on one foot and shifting weight on both feet during the home-based exergame program possibly had a positive effect on improving balance.

The BBS consists of tasks necessary for movement in daily life, and has been used as an evaluation tool to evaluate balance ability and predict falls [33]. The BBS of this study showed a significant improvement of 4.1% from 46.20 points to 48.10 points in the experimental group. This is consistent with a previous study that showed a significant improvement in the BBS score from 50.53 points to 53.93 points after conducting game-based home exercise training for older adults [53]. Cicek et al. [54] also reported a significant improvement in BBS scores after game-based exercise training, suggesting that the home-based exergame program in this study was effective in improving balance.

FRT is a single-task balance test that assesses the maximum distance a person can reach forward while maintaining a fixed base of support in a standing position [34]. Balance ability improved by 11.6% in the experimental group, which was consistent with the results of previous studies showing that video game-based exercise was effective in improving balance ability [24,55]. The home-based exergame program in this study is thought to have contributed to improved balance ability by requiring postural control and weight movement in various directions. In addition, the visual feedback, which enables more efficient and correct movements, seems to have served as an important factor in postural control [56,57].

TUGT includes several factors, such as agility, lower-extremity strength, balance ability, and gait speed [35]. Because TUGT consists of common daily movements, such as standing up from a sitting position, walking, and turning, it provides predictive factors for mobility and balance [31]. In this study, TUGT improved by 12.6% in the experimental group. This is thought to be because the home-based exergame program improved the functional activities and, in turn, promoted interest to participate in physical activities and ultimately maintained postural control. This is consistent with the results of previous studies showing that motivation and interest in exercise led to an awareness of balance control [58].

After the age of 60 years, the number of falls increases by 35–40%, mainly due to decreased muscle strength and balance [13,59,60]. In addition, the lack of physical activity can lead to lower-extremity weakness and balance disorders, which have been found to be important predictors of falls in older adults [13,14]. In this study, the FTSTS evaluated lower-extremity muscle strength, which improved by 19.7% in the experimental group after intervention. In Chen et al. [61] study, older adults who participated in video-based exercise training for 6 weeks showed a 21% improvement in FTSTS. Maillot et al. [62] conducted video-based exercise training for older adults for 12 weeks and showed an improved number of sitting-to-standing movements for 15 s by 21%. Many previous studies have confirmed that video-based exercise programs promote skeletal muscle improvement [63], supporting the results of this study.

Fall experiences and injuries in older adults can easily induce fall phobia, and a fall efficiency evaluation is effective in quantifying phobia [14,15,16]. Many studies have confirmed that a fall experience significantly reduces fall efficiency [64]. Low fall efficacy has a strong influence on the deterioration of walking function, and older adults with low fall efficacy often limit their activity and consequently accelerate the decline in physical function [52]. In this study, the MFES was used to evaluate fall efficacy. In this study, it was confirmed that there was a significant improvement in the experimental group after the 8-week home-based exergame program. Chen et al. [61] conducted video-based exercise training remotely for 6 weeks among older adults and confirmed that the MFES score of the experimental group significantly improved compared to that of the control group. In another study where older adults with Parkinson’s disease performed video-game-based exercises, the MFES score increased significantly [65], which is consistent with this study.

Depression is not a normal aging process but a modifiable risk factor for falls [16]. According to a previous study, the incidence of depression increased by about 2.35 times in people who experienced a fall in the past year compared to those who did not, and depression was found to be closely related to falls [66]. Therefore, it is essential to reduce depression in the older adult population to prevent falls [16,66]. According to a previous study, conducting a remote exergaming program for older adults confirmed that the GDS score significantly decreased [67,68]. In this study, depression was significantly reduced by the home-based exergame program, which is consistent with the results of previous studies.

Quality of life is an important concept in old age, as the satisfaction of daily life is subjectively evaluated. Improved quality of life in older adults can be described as the improvement in emotional state and daily life function by enabling active thinking and action [69]. Since the level of physical activity affects health-related quality of life, it suggests that participation in an exercise program can make a positive change in the quality of life [70]. In this study, physical function, role limitation due to physical health, general health, and vitality were significantly improved in the health-related quality of life evaluation. In a previous study of the exergame program for the older adults, among the SF-36 items, there were significant differences only in physical function, role limitation by physical health, social role functioning, and general health perceptions items [71,72]. As the home-based exergame program in this study consisted of exercises that directly affect physical function, significant improvements were seen in items related to physical function as well.

The limitations of this study include the fact that all participants were from a single community, which may limit the generalizability of the results to other populations. Additionally, the study did not have a long-term follow-up to assess whether the effects of the intervention persisted beyond the 8-week period.

The clinical implication of this study is that home-based exergame programs could be a practical and effective intervention to improve physical function, fall efficacy, depression, and health-related quality of life in community-dwelling older adults. The findings of this study suggest that exergames can be a convenient and accessible exercise option for older adults, particularly those who may face barriers to attending traditional exercise programs in community settings. Healthcare professionals could recommend exergame programs to older adults interested in improving their physical function, fall efficacy, depression, and health-related quality of life.

## 5. Conclusions

Our results suggest that implementing an 8-week home-based exergame program can lead to noteworthy enhancements in physical function, fall efficacy, depression, and health-related quality of life among community-dwelling older adults. Therefore, the advancement and distribution of home-based exergames could have the potential to expand the range of activities and opportunities available to the older adult population.

## Figures and Tables

**Table 1:** General characteristics of the subjects.

	Experimental Group (*n* = 28)	Control Group (*n* = 29)	χ^2^/*t*	*p*
Age (year)	80.39 ± 2.57	79.10 ± 3.90	1.467	0.148
Height (cm)	164.36 ± 7.21	161.55 ± 9.97	1.213	0.230
Weight (kg)	61.40 ± 7.51	60.33 ± 8.86	0.493	0.624
BMI (point)	22.71 ± 2.26	23.04 ± 1.99	0.580	0.564
MMSE	25.93 ± 1.25	25.52 ± 1.02	1.365	0.178
Gender (male/female)	17/11	14/15	0.346	0.888

Values are expressed as mean ± standard deviation. The independent *t*-test and chi-squared tests are used to compare the dependent variables between the two groups; BMI, body mass index; MMSE, mini-mental state examination.

**Table 2:** Changes in physical function.

		Experimental Group (*n* = 28)	Control Group (*n* = 29)	*t*	*p*	CI for Difference		Effect Size	MDC
						Lower	Upper		MDC%
OLST (sec)	Pre	28.09 ± 4.33	27.13 ± 6.54	0.652	0.517	-	-	-	-
	Post	36.43 ± 5.50	27.75 ± 7.35	-	-	-	-	-	-
	Pre–Post	8.34 ± 1.91	0.61 ± 4.92	7.765	0.000	5.73	9.72	2.06	1.00
	*t*	23.149	0.672	-	-	-	-	-	11.97
	*p*	0.000	0.507	-	-	-	-	-	-
BBS (point)	Pre	46.36 ± 4.29	44.62 ± 4.81	1.438	0.156	-	-	-	-
	Post	48.18 ± 3.71	44.86 ± 4.70	-	-	-	-	-	-
	Pre–Post	1.82 ± 1.52	0.24 ± 0.79	4.963	0.000	0.94	2.22	1.31	0.79
	*t*	6.355	1.655	-	-	-	-	-	43.62
	*p*	0.000	0.109	-	-	-	-	-	-
FRT (cm)	Pre	23.29 ± 4.48	24.66 ± 2.75	1.399	0.168	-	-	-	-
	Post	26.57 ± 3.79	24.52 ± 2.76	-	-	-	-	-	-
	Pre–Post	3.28 ± 4.18	−0.14 ± 3.60	3.317	0.002	1.36	5.49	0.88	2.19
	*t*	4.148	0.215	-	-	-	-	-	66.82
	*p*	0.000	0.831	-	-	-	-	-	-
TUGT (sec)	Pre	12.35 ± 3.79	12.18 ± 4.06	0.158	0.875	-	-	-	-
	Post	10.80 ± 3.87	11.97 ± 4.56	-	-	-	-	-	-
	Pre–Post	−1.55 ± 1.17	−0.21 ± 0.90	4.856	0.000	−1.89	−0.79	−1.29	0.00
	*t*	7.014	1.267	-	-	-	-	-	0.00
	*p*	0.000	0.216	-	-	-	-	-	-
FTSTS (sec)	Pre	15.87 ± 4.33	15.73 ± 4.36	0.117	0.908	-	-	-	-
	Post	13.09 ± 3.83	15.94 ± 4.39	-	-	-	-	-	-
	Pre–Post	−2.78 ± 1.76	0.20 ± 2.12	5.770	0.000	−4.02	−1.95	−1.53	0.92
	*t*	8.365	0.519	-	-	-	-	-	33.14
	*p*	0.000	0.608	-	-	-	-	-	-

Values are expressed as mean ± standard deviation. OLST, one-leg standing test; BBS, Berg balance scale; FRT, functional reach test; TUGT, timed up-and-go Test; FTSTS, five-times sit-to-stand test; CI, confidence interval; MDC, minimal detectable change.

**Table 3:** Changes in fall efficacy and depression.

		Experimental Group (*n* = 28)	Control Group (*n* = 29)	*t*	*p*	CI for Difference		Effect Size	MDC
						Lower	Upper		MDC%
MFES (point)	Pre	6.63 ± 1.59	6.07 ±1.70	1.299	0.199	-	-	-	-
	Post	7.05 ± 1.46	5.97 ± 1.50	-	-	-	-	-	-
	Pre–Post	0.42 ± 0.41	−0.10 ± 0.88	2.840	0.006	0.15	0.89	0.75	0.21
	*t*	5.488	0.609	-	-	-	-	-	50.51
	*p*	0.000	0.548	-	-	-	-	-	-
GDS (point)	Pre	13.21 ± 4.52	13.34 ± 4.75	0.106	0.916	-	-	-	-
	Post	10.54 ± 3.84	13.55 ± 4.62	-	-	-	-	-	-
	Pre–Post	−2.68 ± 1.93	0.21 ± 0.94	7.228	0.000	−3.69	−2.09	−1.92	1.01
	*t*	7.361	1.186	-	-	-	-	-	37.66
	*p*	0.000	0.246	-	-	-	-	-	-

Values are expressed as mean ± standard deviation. MFES, modified falls efficacy scale; GDS, geriatric depression scale; CI, confidence interval; MDC, minimal detectable change.

**Table 4:** Changes in health-related quality of life.

		Experimental Group (*n* = 28)	Control Group (*n* = 29)	*t*	*p*	CI for Difference		Effect Size	MDC
						Lower	Upper		MDC%
Physical Function (%)	Pre	62.74 ± 14.91	61.38 ± 14.38	0.350	0.727	-	-	-	-
	Post	65.89 ± 13.31	60.94 ± 15.25	-	-	-	-	-	-
	Pre–Post	3.15 ± 3.35	−0.44 ± 1.94	4.975	0.000	0.02	0.05	1.32	0.02
	*t*	4.985	1.212	-	-	-	-	-	55.60
	*p*	0.000	0.236	-	-	-	-	-	-
Role limitations due to physical health (%)	Pre	58.5% ± 20.27	61.62 ± 16.61	0.536	3.069	-	-	-	-
	Post	61.32 ± 18.00	61.55 ± 17.33	-	-	-	-	-	-
	Pre–Post	2.75 ± 4.46	−0.07 ± 2.10	3.069	0.003	0.01	0.05	0.81	0.02
	*t*	3.262	0.177	-	-	-	-	-	84.97
	*p*	0.003	0.861	-	-	-	-	-	-
Bodily Pain (%)	Pre	61.20 ± 16.62	57.61 ± 16.22	0.824	0.413	-	-	-	-
	Post	61.43 ± 16.37	56.81 ± 17.21	-	-	-	-	-	-
	Pre–Post	0.23 ± 3.57	−0.80 ± 2.61	1.247	0.218	−0.01	0.03	0.33	0.02
	*t*	0.341	1.653	-	-	-	-	-	811.99
	*p*	0.735	0.110	-	-	-	-	-	-
General Health (%)	Pre	54.57 ± 12.71	56.83 ± 12.93	0.664	0.509	-	-	-	-
	Post	56.34 ± 12.48	56.75 ± 12.78	-	-	-	-	-	-
	Pre–Post	1.77 ± 3.58	−0.08 ± 1.02	2.668	0.010	0.00	0.03	0.71	0.02
	*t*	2.615	0.406	-	-	-	-	-	105.99
	*p*	0.014	0.688	-	-	-	-	-	-
Vitality (%)	Pre	61.96 ± 12.27	60.86 ± 12.18	0.340	0.735	-	-	-	-
	Post	62.79 ± 11.92	60.52 ± 12.44	-	-	-	-	-	-
	Pre–Post	0.82 ± 1.72	−0.34 ± 0.94	3.191	0.002	0.00	0.02	0.85	0.00
	*t*	2.523	1.986	-	-	-	-	-	0.00
	*p*	0.018	0.057	-	-	-	-	-	-
Social Functioning (%)	Pre	79.29 ± 17.83	76.90 ± 20.20	0.473	0.638	-	-	-	-
	Post	79.68 ± 17.34	76.90 ± 20.10	-	-	-	-	-	-
	Pre–Post	0.39 ± 2.38	0.00 ± 0.60	0.862	0.392	−0.01	0.01	0.23	0.01
	*t*	0.874	0.000	-	-	-	-	-	317.08
	*p*	0.390	1.000	-	-	-	-	-	-
Role limitations due to emotional problems (%)	Pre	63.57 ± 22.84	62.07 ± 22.26	0.251	0.802	-	-	-	-
	Post	64.18 ± 22.06	62.32 ± 21.89	-	-	-	-	-	-
	Pre–Post	0.61 ± 2.21	0.25 ± 1.46	0.718	0.476	−0.01	0.01	0.19	0.01
	*t*	1.457	0.935	-	-	-	-	-	190.29
	*p*	0.157	0.358	-	-	-	-	-	-
Mental Health (%)	Pre	65.14 ± 12.64	67.17 ± 12.16	0.618	0.539	-	-	-	-
	Post	65.39 ± 12.69	67.52 ± 11.88	-	-	-	-	-	-
	Pre–Post	0.25 ± 0.70	0.34 ± 2.39	0.201	0.841	−0.01	0.01	−0.05	0.00
	*t*	1.888	0.776	-	-	-	-	-	0.00
	*p*	0.070	0.445	-	-	-	-	-	-

Values are expressed as mean ± standard deviation. CI, confidence interval; MDC, minimal detectable change.

## Data Availability

Not applicable.
